# Truncated *O*-glycosylation in metastatic triple-negative breast cancer reveals a gene expression signature associated with extracellular matrix and proteolysis

**DOI:** 10.1038/s41598-024-52204-2

**Published:** 2024-01-20

**Authors:** María Florencia Festari, Eugenio Jara, Monique Costa, Andrés Iriarte, Teresa Freire

**Affiliations:** 1 Laboratorio de Inmunomodulación y Vacunas, Departamento de Inmunobiología, Facultad de Medicina, Universidad de la República, Gral. Flores 2125, 11800 Montevideo, Uruguay; 2 Unidad de Genética y Mejora Animal, Departamento de Producción Animal, Facultad de Veterinaria, Universidad de la República, Montevideo, Uruguay; 3 Laboratorio de Biología Computacional, Departamento de Desarrollo Biotecnológico, Instituto de Higiene, Facultad de Medicina, Universidad de la República, Dr. Alfredo Navarro 3051, 11600 Montevideo, Uruguay

**Keywords:** Cancer, Computational biology and bioinformatics

## Abstract

Breast cancer (BC) is the leading cause of death by cancer in women worldwide. Triple-negative (TN) BC constitutes aggressive and highly metastatic tumors associated with shorter overall survival of patients compared to other BC subtypes. The Tn antigen, a glycoconjugated structure resulting from an incomplete *O*-glycosylation process, is highly expressed in different adenocarcinomas, including BC. It also favors cancer growth, immunoregulation, and metastasis in TNBC. This work describes the differentially expressed genes (DEGs) associated with BC aggressiveness and metastasis in an incomplete *O*-glycosylated TNBC cell model. We studied the transcriptome of a TNBC model constituted by the metastatic murine 4T1 cell line that overexpresses the Tn antigen due to a mutation in one of the steps of the *O*-glycosylation pathway. We analyzed and compared the results with the parental wild-type cell line and with a Tn-negative cell clone that was poorly metastatic and less aggressive than the 4T1 parental cell line. To gain insight into the generated expression data, we performed a gene set analysis. Biological processes associated with cancer development and metastasis, immune evasion, and leukocyte recruitment were highly enriched among functional terms of DEGs. Furthermore, different highly *O*-glycosylated protein-coding genes, such as *mmp9*, *ecm1* and *ankyrin-2*, were upregulated in 4T1/Tn^+^ tumor cells. The altered biological processes and DEGs that promote tumor growth, invasion and immunomodulation might explain the aggressive properties of 4T1/Tn^+^ tumor cells. These results support the hypothesis that incomplete *O*-glycosylation that leads to the expression of the Tn antigen, which might regulate activity or interaction of different molecules, promotes cancer development and immunoregulation.

## Introduction

Cancer develops through an accumulation of genetic or epigenetic alterations that affect key genes with critical roles in the transformation of normal to tumor cells^1^. This occurs throughout a complex and multiple-step process of carcinogenesis across many years involving the inactivation of tumor suppressors or activation of oncogenes^2^. Breast cancer (BC) is the leading cause of death by cancer in women worldwide^3^. Despite early diagnosis and treatments, such as surgery and targeted therapies, the morbidity and mortality caused by BC persist in female patients, with metastasis being the main cause of death^4^. The triple-negative BC (TNBC) subtype, accounting for 15–20% of breast cancers, is characterized by the absence of estrogen and progesterone receptors and Her2 expression^4^. Therefore, there are no approved targeted therapies so far. TNBC is characterized by heterogeneous, aggressive, and highly metastatic tumors that lead to poor prognosis and shorter overall survival of patients when compared to other BC subtypes^4,5^.

Aberrant glycosylation is a hallmark of epithelial cancers, being the result of a series of alterations in the glycosylation pathways of proteins or lipids^6,7^. The expression of carbohydrate tumor-associated antigens favors tumor progression, spreading, and invasiveness of cancer cells^8^. Among these antigens, the Tn antigen (GalNAc-*O*-Thr/Ser) is highly and widely expressed in different adenocarcinomas while absent in normal tissues, and its expression correlates with decreased survival of cancer patients and high metastatic potential of tumor cells^9,10^. The core 1 beta 3-galactosyltransferase is the enzyme that elongates the Tn antigen^11^ and COSMC (core 1 β3Galactosyltransferase specific molecular chaperone) is its chaperone^12^. Previous studies showed that mutations in *cosmc* are associated with metastases and the progression of various types of cancer^13,14^. Therefore, the expression of Tn antigen and core 1 β-3Galactosyltransferase activity loss can result from *cosmc* mutation^12,15–19^. Interestingly, we have previously shown that the Tn antigen favors cancer growth, immunoregulation and metastasis both in lung cancer^20^ and TNBC^21^. We generated a Tn-expressing cell clone from the 4T1 TNBC mouse cell line by targeting *cosmc* with CRISPR/Cas9 gene editing, and demonstrated that the presence of the Tn antigen enhances breast tumor growth and lung metastasis development^21^ in the 4T1 orthotopic TNBC murine cancer model. We also obtained a Tn-negative cell clone (4T1/Tn^−^) that was poorly metastatic and less aggressive than the 4T1 parental cell line^21^.

This work aims to study and to compare the gene expression profiles among the three cell lines to elucidate the regulatory changes that explain the observed invasive and metastatic behavior in the Tn^+^ cell clone. Transcriptomic analyses were used to identify significantly differentially expressed genes (DEGs) involved in biological processes associated with cancer development, metastasis and leukocyte recruitment. Furthermore, this study reports that different highly *O*-glycosylated protein-coding genes, such as *mmp9, ecm1,* and *ankyrin-2* were upregulated in 4T1/Tn^+^ tumor cells. We discuss the obtained results in the context of cell malignancy and incomplete *O*-glycosylation.

## Results and discussion

This work explored differential gene expression between three murine TNBC cell lines: 4T1/wt, 4T1/Tn^+^ and 4T1/Tn^−^, previously characterized by our group^21^. Due to the genetic modification of the *cosmc* gene in the 4T1/Tn^+^ cell line, this model is of great relevance for the study of the significance of truncated cancer-associated *O*-glycans, in particular of the Tn antigen, in the development and malignant behavior of this type of cancer. We did a stranded sequencing of mRNAs in quadruplicate for each cell line. Reads and metadata can be assessed in the NCBI Bioproject database under the accession number Bioproject PRJNA1021590. The 4T1/Tn^+^ cell line was shown to possess higher metastatic potential than the parental 4T1 cell line, while the 4T1/Tn^−^ is not metastatic in the same tested conditions^21^.

### Overall RNA-seq transcriptome results

A total of 752,024,976 (53.3G) raw reads were obtained for all samples. After adapter removal and cleaning by quality and length, 612,675,967 high quality reads were retained for subsequent analyses. A summary of sequencing statistics and mapping rate is presented in Table 1. We mapped these reads to the *Mus musculus* reference genome GRCm38 and obtained an overall unique mapping rate above 95.3 in all samples (Table 1), that is more than 32.8 × 10^6^ concordantly mapped paired reads for each replicate, which is enough to accurately quantify the expression level of genes in eukaryotes (see for instance^22)^. We detected a total of 57,439 annotated genes and novel transcripts. After filtering by gene expression, 21,661 genes and novel transcripts were kept for subsequent analyses. A principal component analysis (PCA) was done in order to study the overall variability between samples under study. The scatter plot in Supplementary Fig. 1 shows the distribution of the 12 samples in the space formed by axis x, the first component, and by axis y, the second component generated by PCA (Supplementary Fig. 1). As can be seen, the replicates from the same cell line cluster together and apart from samples from other cell lines. Note that the first dimension, that explains 58% of the variability, contributes to separating 4T1/Tn^+^ from the other two cell lines, while the second component, explaining 32% of the variability, separates 4T1/wt from 4T1/Tn^+^ and 4T1/Tn^−^. Taken together these results support the robustness of the study and show clear expression differences between analyzed cell lines.

**Table 1:** Basic metrics of the RNA-seq transcriptomic analysis*.

	Raw reads^(MM)^	Clean reads^(MM)^	Mapped reads^(MM)^	Concordantly mapped pairs^(MM)^	Uniquely mapped reads^(%)^
4T1/wt a	75.5	50.4	57.4	23.9	96.7
4T1/wt b	59.9	35.0	41.9	16.4	96.5
4T1/wt c	64.8	42.6	48.8	20.2	96.9
4T1/wt d	74.6	50.5	56.8	23.4	96.2
4T1/Tn^−^ e	55.8	42.8	45.0	19.9	95.8
4T1/Tn^−^ f	59.4	45.2	47.7	21.0	96.1
4T1/Tn^−^ g	66.6	50.7	53.1	22.9	95.3
4T1/Tn^−^ h	58.2	44.8	47.2	20.9	96.2
4T1/Tn^+^ i	71.6	54.8	57.9	25.4	96.3
4T1/Tn^+^ j	50.9	39.2	41.3	18.4	96.5
4T1/Tn^+^ k	62.4	47.6	50.2	21.9	96.1
4T1/Tn^+^ l	52.3	40.0	42.5	18.8	96.7

*MM = million.

### Differential gene expression analysis between TNBC cell lines

**Figure 1:**
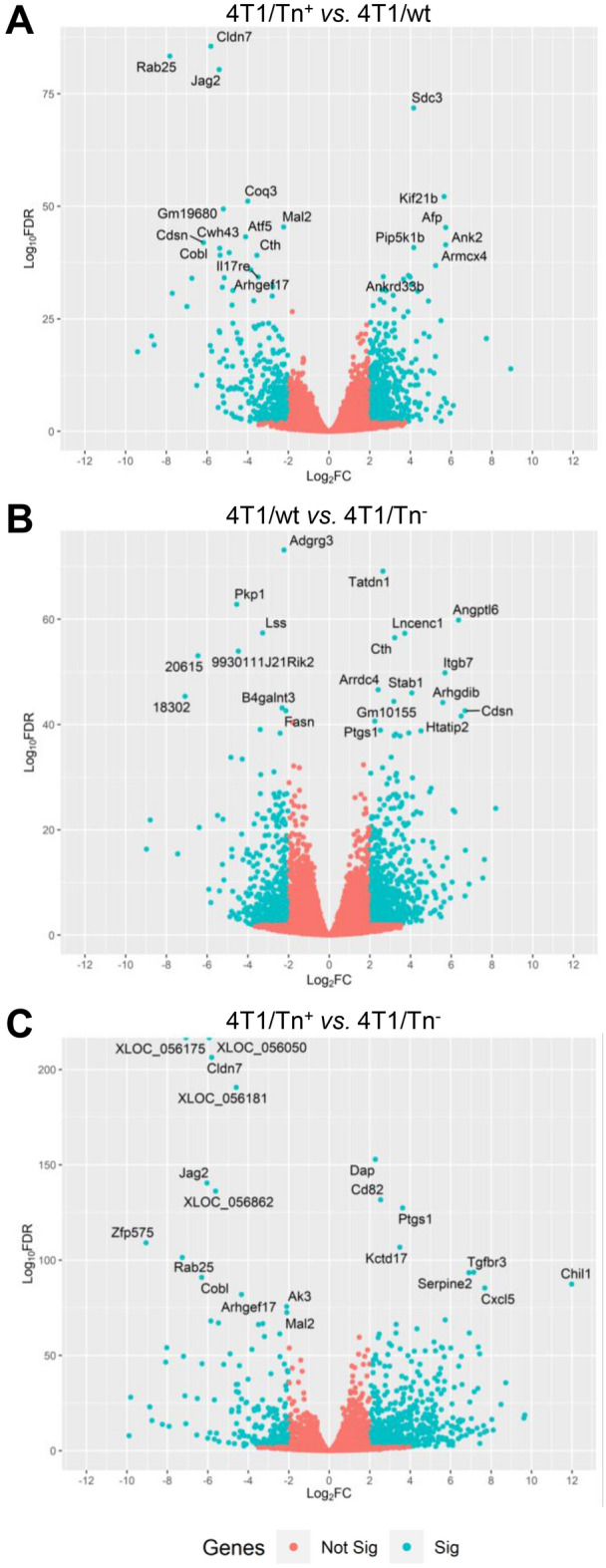
“Volcano plots” of adjusted statistical significance (FDR) against log2 fold change observed in genes in the expression pairwise comparison between analyzed cell lines. DEGs are indicated in blue. Thresholds of significance (< 0.01 FDR) and log2 fold change (> 2). The name of the most significant genes is indicated.

We detected 1405 DEGs between 4T1/Tn^+^ and 4T1/wt and 838 DEGs between 4T1/Tn^+^ and 4T1/Tn^−^ (FDR ≤ 0.01 and |log2FC|≥ 2). Among the DEGs identified between 4T1/Tn^+^ and 4T1/wt, 733 were up-regulated and 672 down-regulated in 4T1/Tn^+^ (Fig. 1 and Supplementary Fig. 2). On the other hand, the differential expression analysis between 4T1/Tn^+^ and 4T1/Tn^−^ showed 623 DEGs up-regulated and 215 down-regulated in 4T1/Tn^+^ (Supplementary Table 1). In addition, we detected a total of 1264 DEGs between 4T1/Tn^−^ and 4T1/wt, 498 genes were up-regulated and 766 were down-regulated in 4T1/wt (Supplementary Table 1).

**Figure 2:**
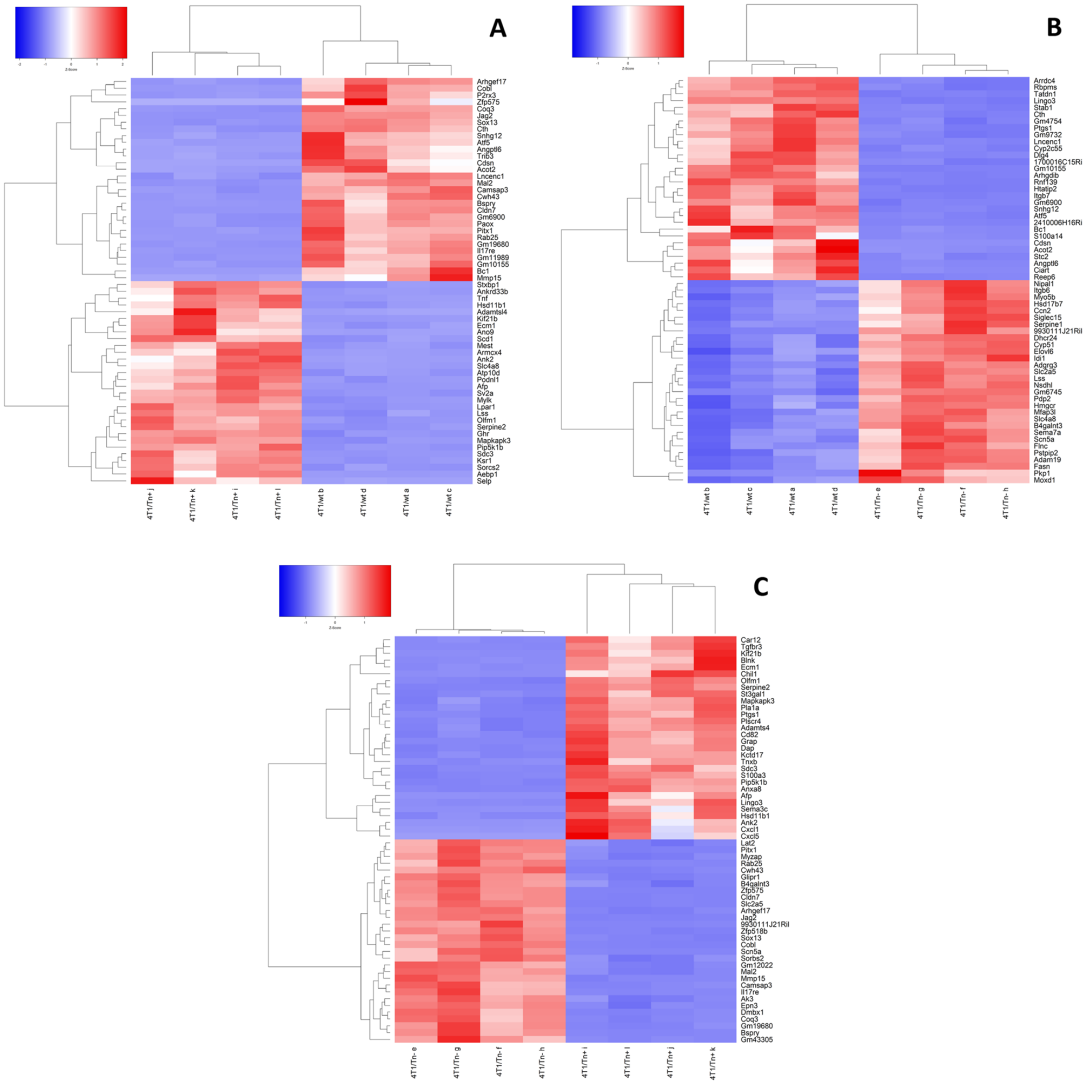
Heatmaps of the top 30 differentially expressed genes (DEGs) identified in each pairwise comparison: (**A**) 4T1/wt versus 4T1/Tn^+^, (**B**) 4T1/wt versus 4T1/Tn^−^, and (**C**) 4T1/Tn^−^ versus 4T1/Tn^+^. Top DEGs are those with the lower FDR values. Genes and samples were grouped by hierarchical clustering.

Firstly, we focused on the most significant up-regulated and downregulated DEGs between all pairwise comparisons (Figs. 1, and 2). Some of these DEGs were up-regulated in 4T1/Tn^+^ when compared to both 4T1/wt and 4T1/Tn^−^ cells and shown to be relevant in cancer: *sdc3*^23^, *kif21b*^24^, *afp*^25^, *ank2*^26^, *pip5k1b*^27^, *serpine2*^28^, *ecm1*^29–31^ and *hsd11b1*^32^. Interestingly, some of these genes encode for *O*-glycosylated proteins, as predicted by the high number of potential *O*-glycosylation sites using the algorithm Net-*O*-Glyc 4.0. On the other hand, *cldn7*, *rab25*, *jag2*, *mal2*, arhgef*17*, *pitx1*, *sox13*, *camsap3* and *mmp15* were down-regulated in 4T1/Tn^+^ cells in relation with 4T1/wt and 4T1/Tn^−^ cells (Fig. 2). It is likely that these DEGs are involved in the specific phenotypic expression and regulation of functional characteristics of 4T1/Tn^+^. Importantly, the transcriptomic data were validated by quantitative real-time PCR (qPCR) of 5 DEGs, that were previously reported as relevant for cancer development (Supplementary Fig. 3): *ecm1*, *serpine2*_,_ *kif21b, camsap3* and *mmp15*.

### Biological processes associated to DEGs in TNBC with truncated *O*-glycosylation

**Figure 3:**
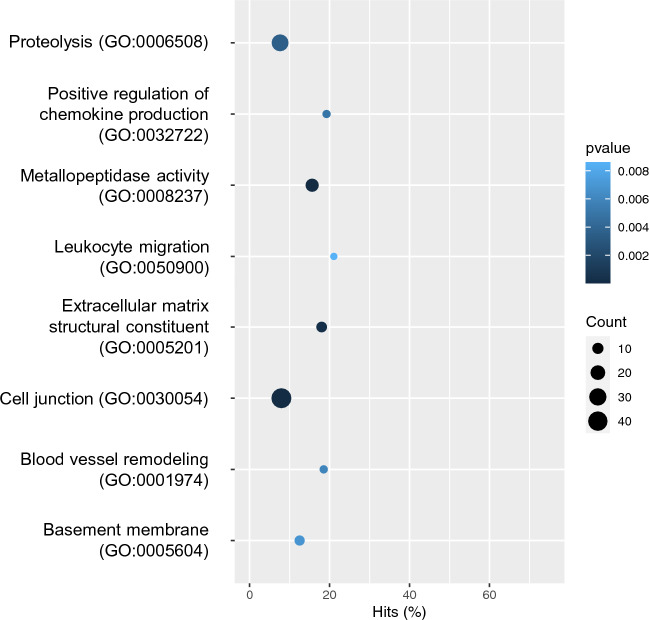
Functional enrichment test results for the functional GO terms mentioned in the main text. The *p*-value of Fisher’s exact test, the number of DEGs, and the percentage of DEGs in the total number of genes are indicated for each GO term.

To highlight the physiological processes that could be related to malignant behavior in TNBC with incomplete *O*-glycosylation, we performed Gene Ontology (GO) enrichment analysis of all DEGs identified in the pairwise comparison of all cell lines. To this end, we focused on the specific patterns of 4T1/Tn^+^ by comparing 4T1/Tn^+^ versus 4T1/wt and 4T1/Tn^+^ versus 4T1/Tn^−^. The GO enrichment analysis showed that functional terms such as *extracellular matrix* (ECM) *structural constituent* (GO:0005201), *cell junction* (GO:0030054), *metallopeptidase activity* (GO:0008237), *basement membrane* (GO:0005604), and *proteolysis* (GO:0006508) (Fig. 3 and Supplementary Table 2), all molecular functions linked to cell invasion and metastasis, were significantly enriched among DEGs. Of note, these biological processes were selected for having more associated DEGs. In addition, immune-related processes, such as *chemokine production* (GO:0032722), *leukocyte migration* (GO:0050900) and *blood vessel remodeling* (GO:0001974), were also selected for their possible connection with aggressiveness of the truncated *O*-glycosylated cell line (Fig. 3 and Supplementary Table 2).

In cancer, changes in the ECM can contribute to tumor growth and metastasis^33^. Several of the obtained enriched GO terms were linked to this process (Supplementary Table 2 and Fig. 3). In addition, we found *ecm1,* an ECM *O*-glycoprotein that interacts with different proteins, maintaining the integrity and homeostasis of skin^34^ and connective tissues^35^, as one of the most up-regulated genes in the 4T1/Tn^+^ cell line. ECM1 is up-regulated in various types of malignant epithelial tumors, including invasive breast ductal carcinoma^36^. In addition, it is a diagnostic marker and correlates with poor prognosis in malignancy of BC^37^. ECM1 induces tumor proliferation, metastasis, epithelial-to-mesenchymal transition (EMT) and blood vessel generation in BC^31,36–38^. Interestingly, this protein is *O*-glycosylated, with five confirmed sites in the N-terminal domain^39^ and one in the C-terminal domain^40^. These sites are among the 27 *O*-glycosylation potential sites predicted by the Net-*O*-Glyc 4.0 algorithm, which are distributed all over the protein. Although the function and structure of these *O*-glycans in cancer progression are still unknown, and considering that ECM1 functions are mediated by interactions with other molecules, it is likely that some of these are affected by the presence of *O*-glycans. Thus, the expression of the Tn antigen in the 4T1/Tn^+^ cell line may regulate these interactions and modulate ECM1 protumor functions. In fact, there are previous works that report that specific *O*-glycan expression modulates protein interactions^39,41^.

Cancer cells can modify the ECM by secreting enzymes, including matrix metalloproteinases (MMPs) and disintegrin and metalloproteinase with thrombospondin motifs (ADAMTS), which were identified in the GO term *metallopeptidase activity* (GO:0008237) and *proteolysis* (GO:0006508). These enzymes degrade various proteins in the ECM allowing the cells to invade surrounding tissues and form new blood vessels that support tumor growth^42,43^. In addition, MMPs can target receptors on the surface of tumor cells, activating pathways that foster proliferation, suppress apoptosis, stimulate metabolic changes or induce EMT^44–46^. Several DEGs that were functionally linked to the GO term *metallopeptidase activity* (GO:0008237) participate in the development of BC and promote tumor progression and angiogenesis, such as *mmp1a*^47^*, mmp9*^48^*, mmp13*^49^ and *mmp15*^50^*.* Interestingly, overexpression of MMP-1 and MMP-9 is crucial in invasion, vascular intravasation, EMT and metastasis of TNBC^51,52^ and are associated with poorer BC patient survival^49^.

MMP-9 is upregulated in invasive BC and is associated with triple-negativity^48^, poor prognosis^53,54^ and metastases^48,55^ in BC patients. Interestingly, MMP-9 has been proposed as a therapeutic target for metastatic breast cancer using 4T1 cells^56^. In the context of this work, it is worth noting the importance of *O*-glycosylation in the regulation of MMPs. Several MMPs have a linker domain that is situated between the Zn^2+^-binding domain and the haemopexin domain^57^. In MMP-9 the linker domain is rich in the amino acids serine, threonine and proline, and was found to be highly *O*-glycosylated^58^. Around 85% of the MMP-9 sugars are *O*-linked and attached to 14 *O*-glycosylation sites^59^. In addition, the membrane anchors and the cytoplasmic domains of the membrane-type MMPs also have potential *O*-glycosylated sites^59^. It has been suggested that, in general, *O*-glycosylation might stabilize MMPs, improving secretion and increasing its protection against degradation. Protection against proteolysis might be important for MMP-9 because it is released at inflammatory sites, where other proteases are likely to be abundant^59^. Cancer-associated glycans expressed on MMP-9 regulate interactions with carbohydrate binding proteins, such as galectins^60^. In the tumor microenvironment, altered glycosylation of MMP-9 allows the detachment of tumor cells from the ECM^61^. The *O*-glycosylated domain also controls the bioavailability of active MMP-9, together with the haemopexin domain, and gives interdomain flexibility to the MMP-9 molecule^58^. This flexibility is important for finding cleavage sites on long substrates^62^. It has also been shown that *O*-glycosylation of MMPs can affect their recruitment to the cell surface^63^, their internalization^64^ or regulate their autolysis^65^, and these effects on one member of the family can even affect the activation of other MMP^63^. Thus, it would be interesting to determine if *mmp9* ablation in our Tn^+^ cell line may reverse the increased aggressiveness associated with Tn expression and also to evaluate whether MMP-Tn activity is different from MMP derived from 4T1 wt cells.

Unlike most MMPs, MMP-15 is a member of the membrane-type MMP subfamily, which are expressed at the cell surface rather than secreted in a soluble form^66^. MMP-15 has anti-apoptotic properties in cervical cancer^67^ and is associated with lung cancer aggressiveness^68^. Furthermore, MMP-15 expression is related with poor prognosis^69^ especially at the transcriptional level. Surprisingly, we found that MMP-15 is downregulated in our 4T1/Tn^+^ cell line with regard to the other analyzed cell lines. In agreement with our observations, contrary data have also been reported^70^, suggesting the existence of different roles for MMP-15. For instance, it can mediate anti-tumor processes by cleaving N-cadherin extracellular domain and therefore preventing cell adhesion^71^. However, its role in TNBC is still unknown. Thus, the analysis of MMP-15 protein expression and its prognostic significance in TNBC remains an important area for future investigation.

Cell junctions are cellular elements that establish links either between two cells or between a cell and the ECM. They are crucial for the maintenance of tissue architecture and proper cell behavior, and their dysfunction can contribute to the development and progression of cancer^72^. Indeed, dysregulation of junction genes has been widely reported in BC^72^. Two out of the 26 DEGs in 4T1/Tn^+^ cells belonging to the *cell junction* GO term (GO:0030054) were identified: *ank2* and *cld7*. Ankyrin-2, encoded by the *ank2* gene, which is highly up-regulated in the malignant cell line, is a ubiquitous structural membrane protein^73^. It participates in cell motility, activation, proliferation, contact, and maintenance of specialized membrane domains. Ankyrin-2 promotes proliferation, migration and invasion of cancer cells and it is also involved in drug resistance of cancer cells^73,74^. Ankyrin-2 has 408 potential *O*-glycosylation sites in its 3898 amino acids, raising the question whether Tn affects the expression or function of this protein in BC. Although Ankyrin-2 expression is increased in solid tumors^32^, no differences between breast tumors and normal tissues have been found. Thus, the significance of its upregulation in the 4T1/Tn^+^ cell line should be further investigated. Claudin-7, encoded by the down-regulated *cld7* gene, has been reported to be less expressed in BC specimens than in normal breast samples, a fact that was previously related with a more aggressive behavior of cancer cells^75^. Claudins are the principal sealing proteins of the tight junctions^76^. Loss of Claudin-7 expression is associated with the discohesive architecture typically observed in high-grade lesions, suggesting potential functional roles for Claudin-7 in BC progression^77^ favoring invasion and metastasis. Nevertheless, the Claudin-7 protein does not present any* O*-glycosylation potential sites, suggesting that the loss of this tumor suppressor^75^ might be involved in the aggressiveness of 4T1/Tn^+^ cells.

An increase in microtubule stability^78^ can drive BC metastasis. In differentiated epithelial cells, most microtubules are not anchored to the centrosome. Instead, their minus-ends are stabilized by binding to a family of proteins, including calmodulin-regulated spectrin-associated proteins (CAMPSAPs)^79^, that were identified in the GO term *structural constituent* (GO:0005201). Interestingly, the loss of *campsap3* promotes AKT-dependent EMT by tubulin acetylation^80^. Since the protein fragment of CAMSAP3 that interacts and stabilizes the microtubule minus-end has many potential *O*-glycosylation sites, we hypothesize that this post-translational modification affects or modulates this interaction, even if this gene is down-regulated.

The *basement membrane* GO term (GO:0005604) contained several significant genes, such as *col17a1*. This gene encodes for a transmembrane protein that is involved in cellular adhesion to the underlying ECM. An under expression of *col17a1* has been demonstrated in BC, likely due to DNA methylation and inactivation of p53, and overexpression in samples of BC^81–83^.

Chronic inflammation and immunosuppression are inducers of cancer progression^84^. Many DEGs related to the identified immune processes were upregulated in the 4T1/Tn^+^ cell line. In this study we found that *nos2*, *nod1* and *chil1* were identified in the *chemokine production* GO term (GO:0032722). The inflammation-associated enzyme, inducible nitric oxide synthase (NOS2) promotes angiogenesis and carcinogenesis and predicts poor survival of ER-negative BC^85^. High levels of NOS2 enhance cell motility and invasion of ER-negative BC^86^. On the other hand, Nod1, a member of the NLR family, acts as a sensor for intracellular bacteria by recognizing specific glycopeptides derived from peptidoglycan. Nod1 activation mediates distinct cellular responses including IL-8 release^87^. Last, chitinase 3-like 1 (CHIL1) is highly secreted by stromal cells in TNBC^88^ and promotes tumor proliferation, invasion and angiogenesis in colorectal cancer^89,90^. Other DEGs that participate in the positive regulation of chemokine production and leukocyte migration, such as TNFα, were also upregulated in 4T1/Tn^+^. The inflammatory cytokine TNFα promotes survival^91^, metastasis^92,93^, EMT^94^, angiogenesis, aggressiveness^95^ and tumor-promoting macrophage infiltration.

### Transcription factor analysis

**Figure 4:**
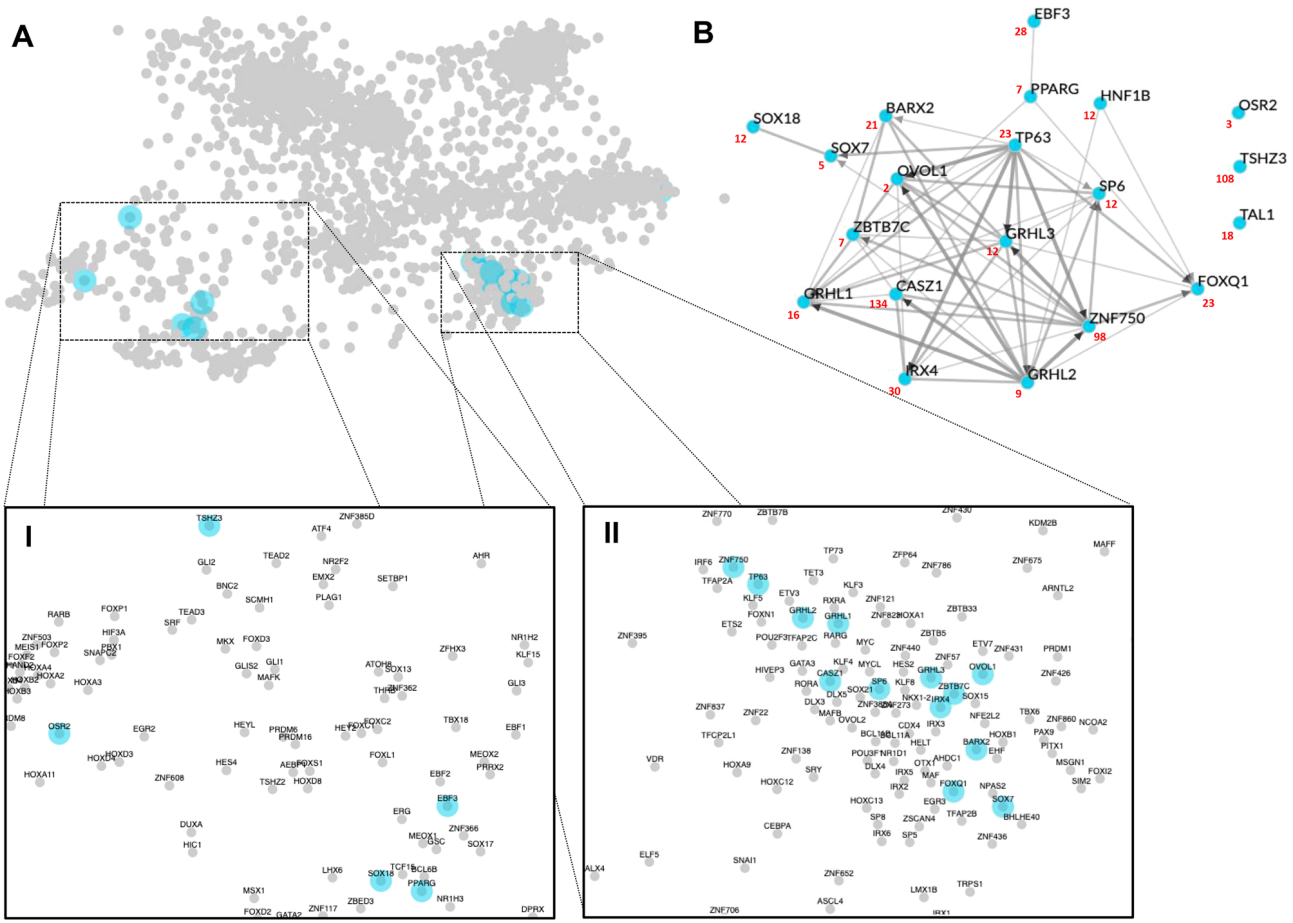
Transcription factor enrichment analysis. (**A**) The scatter plot of human transcription factors (TFs) (each point) was build based on their co-expression similarity. The top 20 enriched TFs are indicated in skyblue. This plot was generated in the ChEA server with default parameters. The WGCNA was done using expression data from GTEx samples and visualized using Cytoscape. (**B**) The local network shows the co-expression similarity among the top 20 identified enriched TFs.

Altered glycosylation patterns on cell surface molecules, transmembrane proteins, and growth factors lead to tumor cell proliferation, invasion, and metastasis through the activation of signaling pathways that can shape the expression of transcription factors (TFs). We identified 386 significantly differentially expressed TF genes (FDR < 0.01 & logFC >|2|) that showed the same trend of expression in 4T1/Tn^+^ compared to 4T1/Tn^−^ and 4T1/wt. When these genes were submitted to the ChEA3 server, 217 were mapped to orthologs in the human genome that were further considered for analysis (Supplementary Table 3). Based on the mean-rank method, we selected the top 20 TFs that regulated 186 out of the 217 submitted DEGs (Supplementary Table 1). Five TFs clustered in distant regions of the global regulatory network (Fig. 4A, Box I), three of which were enriched in the functional term s*tem cell fate specification* (*ebf3*, *sox18* and *pparg*) (Supplementary Fig. 4). Likewise, thirteen TFs were closely grouped in the generated global network, meaning that they show high co-expression similarity (Fig. 4A, Box II). The functional GO term enrichment analysis suggested that they are mainly linked to the regulation of genes associated with *epidermis development* (*foxq1*, *irx4*, *sp6*, *casz1*, *grhl2*, *tp63*, *znf750*) and *positive regulation of wound healing* (*ovol1*, *barx2*, *sox7*, *zbtb7c*, *grhl1*, *grhl3*) (Supplementary Fig. 4). Both functional processes have been linked to BC growth in a wide variety of functional genomics studies ^96–100^. The local network (Fig. 4B) which included only the 20 identified TFs, indicated that the expression of all but three selected TFs were correlated in the GTEx database, with some TFs showing strong associations (e.g., *grhl3*, *tp63*, *znf750*, *ovol1* and *casz1*) (Fig. 4B). The differential signaling due to altered* O*-glycosylated patterns might explain the presence of a different TF signature in 4T1/Tn^+^ cells, since human BC cells with distinct *O*-linked glycans respond different to EGF binding. This variation is attributed to the differential nuclear translocation of EGFR^101^ and is regulated by the formation of an EGFR/galectin3/MUC1/β-catenin complex at the cell surface, which depends on the *O*-glycan signature of the cells^101^. Therefore, the identified TFs are likely regulated by glycosylation of different proteins involved in signal transduction cascades in the 4T1/Tn^+^ cell line.

### Limitations of this study

As with any research, there are limitations in the study worth mentioning. First, these observations are limited to one TNBC cell line murine model. The transcriptomic study on further TNBC human models will undoubtedly validate the results obtained in the present work. To this end, new TNBC *cosmc* knock out cell derivatives need to be obtained and characterized. Secondly, since the study of in vitro cultured cancer cells allows to determine the transcriptome only from tumor cells, the use of tumor samples is required to reproduce the heterogeneity and behavior of tumors. Indeed, the existence of other cell types in the tumor microenvironment, as cancer-associated fibroblasts and immune cells, can affect the Tn expression in the surface of cancer cells. In this sense, the study of human organoids is an interesting alternative, since they resemble to the complexity of the tumor allowing genetic engineering^102^.

## Conclusions

In this work, we describe the gene expression profile of BC aggressiveness and metastasis in a TNBC preclinical model of truncated *O*-glycosylation. The altered biological processes and DEGs that promote tumor growth, invasion and immunomodulation might explain the aggressive properties of 4T1/Tn^+^ tumor cells^21^. Furthermore, different highly *O*-glycosylated protein coding genes, such as *mmp9*, *ecm1* and *ank2*, were upregulated in 4T1/Tn^+^ tumor cells. These results support the hypothesis that incomplete *O*-glycosylation that leads to the expression of the Tn antigen, which might regulate their activity or interaction of different molecules, promotes cancer development and immunoregulation. The role of *O*-glycans in the identified molecules remains to be elucidated.

## Methods

### Cell culture

The murine TNBC cell line 4T1 was obtained from ATCC and cultured in DMEM with glutamine (Capricorn, Germany or Gibco, USA) supplemented with 10% inactivated fetal bovine serum (Capricorn, Germany) and antibiotic–antimycotic (Thermo Fisher) at a final concentration of 100 units/mL of penicillin, 100 µg/mL of streptomycin, and 0.25 µg/mL of Gibco Amphotericin B (complete culture medium). The 4T1/Tn^+^ cell line was generated by CRISPR/Cas9 guide targeted to *cosmc* gene exon 2 170 s (GATATCTCGAAAATTTCAG) cloned in pBS-U6sg plasmid (Tacgene, France) and GFP-tagged Cas9-PBKS plasmid (Addgene), as previously described^21^. Cells were obtained by flow cytometry sorting (BD FACSAria™ Fusion). The expression of Tn antigen was verified by staining with the anti-Tn mAb 83D4^21^ in the obtained Tn^+^ cell line. A Tn^−^ cell clone was also selected for further characterization according to Tn expression. Cells were maintained at 37 °C in a humidified atmosphere of 5% CO_2_ and  harvested by washing with phosphate-buffered saline (PBS) pH 7.4 and incubation with trypsin (0.1%) and EDTA (0.04%) in PBS.

### RNA isolation and sequencing

RNA from 4 replicates of each cell line, wild-type, Tn^+^ and Tn^−^, was purified using the RNeasy kit from Qiagen according to the instructions of the manufacturer. Both RNA concentration and integrity (RIN values) were measured in an Agilent 2100 Bioanalyzer (Agilent, USA). A total of 4 μg of RNA per sample was used as the input for mRNA library preparations. Twelve libraries were generated using Illumina Truseq Stranded mRNA kit. Then, all libraries were sequenced using NextSeq Illumina platform at Macrogen Inc. (Korea), in one lane, using 150 bp paired-end reads.

### Reads trimming and mapping

Quality control of raw reads were performed using FastQC^103^. Adapter sequences and low-quality reads were removed using scythe v0.991 (github.com/vsbuffalo/scythe) and sickle v1.33 (github.com/najoshi/sickle). High-quality reads were mapped to the *Mus musculus* reference genome (i.e. version GRCm38 downloaded from Ensembl database) using the software Hisat2 (v2.1.0)^104^. Hisat2 was run with ‘--rna-strandness RF’ and ‘-k 1’, with all other parameters set as default.

### Assembly of transcripts and estimation of abundance

Cufflinks (v2.2.1) was used to assemble transcript models from read alignments^105^. This software recreates a set of transcript models that best explain the sequencing alignments observed in the samples. Cufflinks was run with '--library-type fr-firststrand’, with all other parameters set as default, for each sample separately. Then, a single annotation file was generated using the tool Cuffmerge^105^. This software merges each of the sample assemblies with the reference annotation file in order to combine novel transcripts with known annotated transcripts. Finally, for each sample, the total number of reads that effectively mapped to each gene described in the final assembly was calculated using the function feature counts of the library Rsubread^106^. In order to visualize the overall relationship between the samples under study, a PCA analysis was performed using DESeq2 (version 1.18.1)^107^.

### Gene expression and gene enrichment analysis

All pairwise comparisons between cell lines were done, that is, 4T1/wt versus 4T1/Tn^+^, 4T1/wt versus 4T1/Tn^−^, and 4T1/Tn^−^ versus 4T1/Tn^+^. DEGs were identified using the R package DESeq2 (version 1.18.1) with default parameters^107^. Only genes with at least one read count in at least ten samples were considered expressed and used for further analysis. The *P*-values were adjusted for multiple testing using the FDR procedure^108^. Those genes having a FDR ≤ 0.01 and a value of |log2FC|≥ 2 were considered significantly differentially expressed.

### Gene Ontology (GO) enrichment analysis

The functional roles of the DEGs were studied using a GO enrichment analysis. GO terms were downloaded using the Bioconductor R package biomaRt (version 2.48.3)^109^. In detail, the predicted DEGs were compared with all the expressed genes as background sets. GO terms significantly enriched within DEGs were detected using Fisher's exact test using R.

### Transcription factor enrichment analysis (TFEA) and prediction of putative *O*-GalNAc sites

In order to identify putative transcription factors (TFs) involved in the regulatory changes observed in the 4T1/Tn^+^ cell line we used the ChIP-X Enrichment Analysis 3 (ChEA3) software^110^. Significantly DEGs that showed the same expression trend (up-regulated or down-regulated) in the 4T1/Tn^+^ cell line when compared to 4T1/Tn^+^ or 4T1/wt were submitted to the ChEA3 online server, available at maayanlab.cloud/chea3. A list of mouse gene symbols was submitted and automatically mapped to available human orthologs. The Mean-rank method (default) was used to identify the TFs whose putative transcriptional targets were most closely similar to the gene set. Co-expression networks, representing co-expression similarity in humans, were built using Weighted Gene Co-expression Network Analysis (WGCNA)^111^ from expression data from GTEx samples^112^. The Net-*O*-Glyc 4.0 program^113^ was used to predict mucin type *O*-GalNAc glycosylation sites in all expressed proteins, including identified TFs.

### Validation by quantitative real-time RT-PCR (qRT-PCR)

The qPCR assay was performed to validate the reliability of the identified DEGs. Total RNA from the three cell lines was isolated using TRI-reagent (Sigma-Aldrich) according to the instructions of the manufacturer. Samples were analyzed in an Eco real-time PCR System (Illumina) using Fast SYBR® Green Master Mix (Applied Biosystems). The PCR conditions were as follows: 45 °C for 5 min, 94 °C for 30 s, 45 cycles of 94 °C for 5 s, and 60 °C for 34 s. The melt curve of each amplicon was set as 95 °C for 15 s, 60 °C for 1 min, 95 °C for 30 s, and 60 °C for 30 s. The primers used are shown in Supplementary Table 4. Results were expressed as the ratio between each evaluated gene and *gapdh* expression. The relative quantification of all selected genes was evaluated using the 2 − ∆∆CT method and normalized to *gapdh*. All reactions were performed with three biological replicates.

### Supplementary Information

Supplementary Figures.

Supplementary Table 1.

Supplementary Table 2.

Supplementary Table 3.

Supplementary Table 4.

## Data Availability

The datasets generated and analyzed during the current study are available in the NCBI Bioproject database under the accession number Bioproject PRJNA1021590 at https://www.ncbi.nlm.nih.gov/bioproject/PRJNA1021590. Materials are available from the corresponding author on reasonable request.

## References

[1] Hanahan D, Weinberg RA (2000). The hallmarks of cancer. Cell.

[2] Nathanson, S. D. *et al.* Associations amongst genes, molecules, cells, and organs in breast cancer metastasis. *Clin. Exp. Metastasis* 10.1007/s10585-023-10230-w (2023). 10.1007/s10585-023-10230-w 37688650

[3] Ferlay, J., Ervik, M., Lam, F., al. e. Global Cancer Observatory: Cancer Today (GLOBOCAN 2020 version 2.0) Lyon. International Agency for Research on Cancer (IARC). Available from https://gco.iarc.fr/today/home.

[4] Hoxha I, Sadiku F, Hoxha L, Nasim M, Christine Buteau MA, Grezda K (2023). Breast cancer and lifestyle factors: Umbrella review. Hematol. Oncol. Clin. N. Am..

[5] Yin L, Duan JJ, Bian XW, Yu SC (2020). Triple-negative breast cancer molecular subtyping and treatment progress. Breast Cancer Res..

[6] Peixoto A, Relvas-Santos M, Azevedo R, Santos LL, Ferreira JA (2019). Protein glycosylation and tumor microenvironment alterations driving cancer hallmarks. Front. Oncol..

[7] Mereiter S, Balmana M, Campos D, Gomes J, Reis CA (2019). Glycosylation in the era of cancer-targeted therapy: Where are we heading?. Cancer Cell.

[8] da Costa V, Freire T (2022). Advances in the immunomodulatory properties of glycoantigens in cancer. Cancers (Basel).

[9] Berois N, Pittini A, Osinaga E (2022). Targeting tumor glycans for cancer therapy: Successes, limitations, and perspectives. Cancers (Basel).

[10] Liu Z, Liu J, Dong X, Hu X, Jiang Y, Li L (2019). Tn antigen promotes human colorectal cancer metastasis via H-Ras mediated epithelial-mesenchymal transition activation. J. Cell Mol. Med..

[11] Ju T, Brewer K, D'Souza A, Cummings RD, Canfield WM (2002). Cloning and expression of human core 1 beta1,3-galactosyltransferase. J. Biol. Chem..

[12] Ju T, Cummings RD (2002). A unique molecular chaperone Cosmc required for activity of the mammalian core 1 beta 3-galactosyltransferase. Proc. Natl. Acad. Sci. U. S. A..

[13] Wu YM, Liu CH, Huang MJ, Lai HS, Lee PH, Hu RH (2013). C1GALT1 enhances proliferation of hepatocellular carcinoma cells via modulating MET glycosylation and dimerization. Cancer Res..

[14] Lee PC, Chen ST, Kuo TC, Lin TC, Lin MC, Huang J (2020). C1GALT1 is associated with poor survival and promotes soluble Ephrin A1-mediated cell migration through activation of EPHA2 in gastric cancer. Oncogene.

[15] Ju T, Cummings RD (2005). Protein glycosylation: Chaperone mutation in Tn syndrome. Nature.

[16] Ju T, Lanneau GS, Gautam T, Wang Y, Xia B, Stowell SR (2008). Human tumor antigens Tn and sialyl Tn arise from mutations in Cosmc. Cancer Res..

[17] Schietinger A, Philip M, Yoshida BA, Azadi P, Liu H, Meredith SC (2006). A mutant chaperone converts a wild-type protein into a tumor-specific antigen. Science.

[18] Crew VK, Singleton BK, Green C, Parsons SF, Daniels G, Anstee DJ (2008). New mutations in C1GALT1C1 in individuals with Tn positive phenotype. Br. J. Haematol..

[19] Sun X, Ju T, Cummings RD (2018). Differential expression of Cosmc, T-synthase and mucins in Tn-positive colorectal cancers. BMC Cancer.

[20] da Costa V, van Vliet SJ, Carasi P, Frigerio S, Garcia PA, Croci DO (2021). The Tn antigen promotes lung tumor growth by fostering immunosuppression and angiogenesis via interaction with Macrophage Galactose-type lectin 2 (MGL2). Cancer Lett..

[21] Festari MF, da Costa V, Rodriguez-Zraquia SA, Costa M, Landeira M, Lores P (2022). The tumor-associated Tn antigen fosters lung metastasis and recruitment of regulatory T cells in triple negative breast cancer. Glycobiology..

[22] Liu Y, Zhou J, White KP (2014). RNA-seq differential expression studies: more sequence or more replication?. Bioinformatics.

[23] Czarnowski D (2021). Syndecans in cancer: A review of function, expression, prognostic value, and therapeutic significance. Cancer Treat. Res. Commun..

[24] Xu S, Li Y, Huang H, Miao X, Gu Y (2022). Identification of KIF21B as a biomarker for colorectal cancer and associated with poor prognosis. J. Oncol..

[25] Glowska-Ciemny J, Szymanski M, Kuszerska A, Malewski Z, von Kaisenberg C, Kocylowski R (2023). The role of alpha-fetoprotein (AFP) in contemporary oncology: The path from a diagnostic biomarker to an anticancer drug. Int. J. Mol. Sci..

[26] Wang T, Abou-Ouf H, Hegazy SA, Alshalalfa M, Stoletov K, Lewis J (2016). Ankyrin G expression is associated with androgen receptor stability, invasiveness, and lethal outcome in prostate cancer patients. J. Mol. Med. (Berl.).

[27] Yin M, Wang Y (2022). The role of PIP5K1A in cancer development and progression. Med. Oncol..

[28] Sasahira T, Kurihara-Shimomura M, Nishiguchi Y, Shimomura H, Kirita T (2020). Sushi repeat containing protein X-linked 2 is a downstream signal of LEM domain containing 1 and acts as a tumor-promoting factor in oral squamous cell carcinoma. Int. J. Mol. Sci..

[29] Lee KM, Nam K, Oh S, Lim J, Kim RK, Shim D (2015). ECM1 regulates tumor metastasis and CSC-like property through stabilization of beta-catenin. Oncogene.

[30] Long S, Wang J, Weng F, Xiang D, Sun G (2022). Extracellular matrix protein 1 regulates colorectal cancer cell proliferative, migratory, invasive and epithelial-mesenchymal transition activities through the PI3K/AKT/GSK3beta/snail signaling axis. Front. Oncol..

[31] Steinhaeuser SS, Morera E, Budkova Z, Schepsky A, Wang Q, Rolfsson O (2020). ECM1 secreted by HER2-overexpressing breast cancer cells promotes formation of a vascular niche accelerating cancer cell migration and invasion. Lab Investig..

[32] Cirillo N, Morgan DJ, Pedicillo MC, Celentano A, Lo Muzio L, McCullough MJ (2017). Characterisation of the cancer-associated glucocorticoid system: Key role of 11beta-hydroxysteroid dehydrogenase type 2. Br. J. Cancer.

[33] Winkler J, Abisoye-Ogunniyan A, Metcalf KJ, Werb Z (2020). Concepts of extracellular matrix remodelling in tumour progression and metastasis. Nat. Commun..

[34] Sercu S, Lambeir AM, Steenackers E, El Ghalbzouri A, Geentjens K, Sasaki T (2009). ECM1 interacts with fibulin-3 and the beta 3 chain of laminin 332 through its serum albumin subdomain-like 2 domain. Matrix Biol..

[35] Fujimoto N, Terlizzi J, Brittingham R, Fertala A, McGrath JA, Uitto J (2005). Extracellular matrix protein 1 interacts with the domain III of fibulin-1C and 1D variants through its central tandem repeat 2. Biochem. Biophys. Res. Commun..

[36] Wang L, Yu J, Ni J, Xu XM, Wang J, Ning H (2003). Extracellular matrix protein 1 (ECM1) is over-expressed in malignant epithelial tumors. Cancer Lett..

[37] Wu QW, She HQ, Liang J, Huang YF, Yang QM, Yang QL (2012). Expression and clinical significance of extracellular matrix protein 1 and vascular endothelial growth factor-C in lymphatic metastasis of human breast cancer. BMC Cancer.

[38] Lee KM, Nam K, Oh S, Lim J, Kim YP, Lee JW (2014). Extracellular matrix protein 1 regulates cell proliferation and trastuzumab resistance through activation of epidermal growth factor signaling. Breast Cancer Res..

[39] Yang W, Ao M, Hu Y, Li QK, Zhang H (2018). Mapping the O-glycoproteome using site-specific extraction of O-linked glycopeptides (EXoO). Mol. Syst. Biol..

[40] Kawahara R, Ortega F, Rosa-Fernandes L, Guimaraes V, Quina D, Nahas W (2018). Distinct urinary glycoprotein signatures in prostate cancer patients. Oncotarget.

[41] Goth CK, Mehta AY, McQuillan AM, Baker KJ, Hanes MS, Park SS (2023). Chemokine binding to PSGL-1 is controlled by O-glycosylation and tyrosine sulfation. Cell Chem. Biol..

[42] Kumar S, Rao N, Ge R (2012). Emerging roles of ADAMTSs in angiogenesis and cancer. Cancers (Basel).

[43] Kelwick R, Desanlis I, Wheeler GN, Edwards DR (2015). The ADAMTS (a disintegrin and metalloproteinase with thrombospondin motifs) family. Genome Biol..

[44] Radisky ES, Radisky DC (2010). Matrix metalloproteinase-induced epithelial-mesenchymal transition in breast cancer. J. Mammary Gland Biol. Neoplasia.

[45] Nistico P, Bissell MJ, Radisky DC (2012). Epithelial-mesenchymal transition: general principles and pathological relevance with special emphasis on the role of matrix metalloproteinases. Cold Spring Harb. Perspect. Biol..

[46] Orive-Ramos A, Seoane S, Ocana A, Pandiella A, Montero JC (2017). Regulation of the prometastatic neuregulin-MMP13 axis by SRC family kinases: therapeutic implications. Mol. Oncol..

[47] Bostrom P, Soderstrom M, Vahlberg T, Soderstrom KO, Roberts PJ, Carpen O (2011). MMP-1 expression has an independent prognostic value in breast cancer. BMC Cancer.

[48] Yousef EM, Tahir MR, St-Pierre Y, Gaboury LA (2014). MMP-9 expression varies according to molecular subtypes of breast cancer. BMC Cancer.

[49] Zhang B, Cao X, Liu Y, Cao W, Zhang F, Zhang S (2008). Tumor-derived matrix metalloproteinase-13 (MMP-13) correlates with poor prognoses of invasive breast cancer. BMC Cancer.

[50] McGowan PM, Duffy MJ (2008). Matrix metalloproteinase expression and outcome in patients with breast cancer: Analysis of a published database. Ann. Oncol..

[51] Wang QM, Lv L, Tang Y, Zhang L, Wang LF (2019). MMP-1 is overexpressed in triple-negative breast cancer tissues and the knockdown of MMP-1 expression inhibits tumor cell malignant behaviors in vitro. Oncol. Lett..

[52] Mehner C, Hockla A, Miller E, Ran S, Radisky DC, Radisky ES (2014). Tumor cell-produced matrix metalloproteinase 9 (MMP-9) drives malignant progression and metastasis of basal-like triple negative breast cancer. Oncotarget.

[53] Vasaturo F, Solai F, Malacrino C, Nardo T, Vincenzi B, Modesti M (2013). Plasma levels of matrix metalloproteinases 2 and 9 correlate with histological grade in breast cancer patients. Oncol. Lett..

[54] Li H, Qiu Z, Li F, Wang C (2017). The relationship between MMP-2 and MMP-9 expression levels with breast cancer incidence and prognosis. Oncol. Lett..

[55] Min KW, Kim DH, Do SI, Kim K, Lee HJ, Chae SW (2014). Expression patterns of stromal MMP-2 and tumoural MMP-2 and -9 are significant prognostic factors in invasive ductal carcinoma of the breast. APMIS.

[56] Hosseini F, Hassannia H, Mahdian-Shakib A, Jadidi-Niaragh F, Enderami SE, Fattahi M (2017). Targeting of crosstalk between tumor and tumor microenvironment by beta-D mannuronic acid (M2000) in murine breast cancer model. Cancer Med..

[57] Cui N, Hu M, Khalil RA (2017). Biochemical and biological attributes of matrix metalloproteinases. Prog. Mol. Biol. Transl. Sci..

[58] Van den Steen PE, Van Aelst I, Hvidberg V, Piccard H, Fiten P, Jacobsen C (2006). The hemopexin and O-glycosylated domains tune gelatinase B/MMP-9 bioavailability via inhibition and binding to cargo receptors. J. Biol. Chem..

[59] Boon L, Ugarte-Berzal E, Vandooren J, Opdenakker G (2016). Glycosylation of matrix metalloproteases and tissue inhibitors: Present state, challenges and opportunities. Biochem. J..

[60] Boon L, Ugarte-Berzal E, Martens E, Vandooren J, Rybakin V, Colau D (2019). Propeptide glycosylation and galectin-3 binding decrease proteolytic activation of human proMMP-9/progelatinase B. FEBS J..

[61] Fry SA, Van den Steen PE, Royle L, Wormald MR, Leathem AJ, Opdenakker G (2006). Cancer-associated glycoforms of gelatinase B exhibit a decreased level of binding to galectin-3. Biochemistry.

[62] Rosenblum G, Van den Steen PE, Cohen SR, Bitler A, Brand DD, Opdenakker G (2010). Direct visualization of protease action on collagen triple helical structure. PLoS ONE.

[63] Wu YI, Munshi HG, Sen R, Snipas SJ, Salvesen GS, Fridman R (2004). Glycosylation broadens the substrate profile of membrane type 1 matrix metalloproteinase. J. Biol. Chem..

[64] Kim S, Huang W, Mottillo EP, Sohail A, Ham YA, Conley-Lacomb MK (2010). Posttranslational regulation of membrane type 1-matrix metalloproteinase (MT1-MMP) in mouse PTEN null prostate cancer cells: Enhanced surface expression and differential O-glycosylation of MT1-MMP. Biochim. Biophys. Acta.

[65] Remacle AG, Chekanov AV, Golubkov VS, Savinov AY, Rozanov DV, Strongin AY (2006). O-glycosylation regulates autolysis of cellular membrane type-1 matrix metalloproteinase (MT1-MMP). J. Biol. Chem..

[66] Chen L, Zhou Q, Xu B, Liu J, Shi L, Zhu D (2014). MT2-MMP expression associates with tumor progression and angiogenesis in human lung cancer. Int. J. Clin. Exp. Pathol..

[67] Abraham R, Schafer J, Rothe M, Bange J, Knyazev P, Ullrich A (2005). Identification of MMP-15 as an anti-apoptotic factor in cancer cells. J. Biol. Chem..

[68] Pietrzak J, Szmajda-Krygier D, Wosiak A, Swiechowski R, Michalska K, Mirowski M (2022). Changes in the expression of membrane type-matrix metalloproteinases genes (MMP14, MMP15, MMP16, MMP24) during treatment and their potential impact on the survival of patients with non-small cell lung cancer (NSCLC). Biomed. Pharmacother..

[69] Wu Y, Pan S, Leng J, Xie T, Jamal M, Yin Q (2019). The prognostic value of matrix metalloproteinase-7 and matrix metalloproteinase-15 in acute myeloid leukemia. J. Cell Biochem..

[70] Asano T, Tada M, Cheng S, Takemoto N, Kuramae T, Abe M (2008). Prognostic values of matrix metalloproteinase family expression in human colorectal carcinoma. J. Surg. Res..

[71] Kuriyama S, Yoshida M, Yano S, Aiba N, Kohno T, Minamiya Y (2016). LPP inhibits collective cell migration during lung cancer dissemination. Oncogene.

[72] Knights AJ, Funnell AP, Crossley M, Pearson RC (2012). Holding tight: Cell junctions and cancer spread. Trends Cancer Res..

[73] Cao W, Wei W, Zhan Z, Xie D, Xie Y, Xiao Q (2018). Regulation of drug resistance and metastasis of gastric cancer cells via the microRNA647-ANK2 axis. Int. J. Mol. Med..

[74] Savas S, Azorsa DO, Jarjanazi H, Ibrahim-Zada I, Gonzales IM, Arora S (2011). NCI60 cancer cell line panel data and RNAi analysis help identify EAF2 as a modulator of simvastatin and lovastatin response in HCT-116 cells. PLoS ONE.

[75] Wang K, Xu C, Li W, Ding L (2018). Emerging clinical significance of claudin-7 in colorectal cancer: A review. Cancer Manag. Res..

[76] Tsukita S, Furuse M (1999). Occludin and claudins in tight-junction strands: Leading or supporting players?. Trends Cell Biol..

[77] Kominsky SL, Argani P, Korz D, Evron E, Raman V, Garrett E (2003). Loss of the tight junction protein claudin-7 correlates with histological grade in both ductal carcinoma in situ and invasive ductal carcinoma of the breast. Oncogene.

[78] Boggs AE, Vitolo MI, Whipple RA, Charpentier MS, Goloubeva OG, Ioffe OB (2015). alpha-Tubulin acetylation elevated in metastatic and basal-like breast cancer cells promotes microtentacle formation, adhesion, and invasive migration. Cancer Res..

[79] Hendershott MC, Vale RD (2014). Regulation of microtubule minus-end dynamics by CAMSAPs and patronin. Proc. Natl. Acad. Sci. U. S. A..

[80] Pongrakhananon V, Wattanathamsan O, Takeichi M, Chetprayoon P, Chanvorachote P (2018). Loss of CAMSAP3 promotes EMT via the modification of microtubule-Akt machinery. J. Cell Sci..

[81] Yodsurang V, Tanikawa C, Miyamoto T, Lo PHY, Hirata M, Matsuda K (2017). Identification of a novel p53 target, COL17A1, that inhibits breast cancer cell migration and invasion. Oncotarget.

[82] Yan X, Zhang C, Liang T, Yang F, Wang H, Wu F (2017). A PTEN-COL17A1 fusion gene and its novel regulatory role in collagen XVII expression and GBM malignance. Oncotarget.

[83] Thangavelu PU, Krenacs T, Dray E, Duijf PH (2016). In epithelial cancers, aberrant COL17A1 promoter methylation predicts its misexpression and increased invasion. Clin. Epigenet..

[84] Zhao H, Wu L, Yan G, Chen Y, Zhou M, Wu Y (2021). Inflammation and tumor progression: signaling pathways and targeted intervention. Signal Transduct. Target. Ther..

[85] Heinecke JL, Ridnour LA, Cheng RY, Switzer CH, Lizardo MM, Khanna C (2014). Tumor microenvironment-based feed-forward regulation of NOS2 in breast cancer progression. Proc. Natl. Acad. Sci. U. S. A..

[86] Glynn SA, Boersma BJ, Dorsey TH, Yi M, Yfantis HG, Ridnour LA (2010). Increased NOS2 predicts poor survival in estrogen receptor-negative breast cancer patients. J. Clin. Investig..

[87] da Silva CJ, Miranda Y, Leonard N, Hsu J, Ulevitch RJ (2007). Regulation of Nod1-mediated signaling pathways. Cell Death Differ..

[88] Malone MK, Smrekar K, Park S, Blakely B, Walter A, Nasta N (2020). Cytokines secreted by stromal cells in TNBC microenvironment as potential targets for cancer therapy. Cancer Biol. Ther..

[89] Kawada M, Seno H, Kanda K, Nakanishi Y, Akitake R, Komekado H (2012). Chitinase 3-like 1 promotes macrophage recruitment and angiogenesis in colorectal cancer. Oncogene.

[90] Watanabe K, Shiga K, Maeda A, Harata S, Yanagita T, Suzuki T (2022). Chitinase 3-like 1 secreted from cancer-associated fibroblasts promotes tumor angiogenesis via interleukin-8 secretion in colorectal cancer. Int. J. Oncol..

[91] Pileczki V, Braicu C, Gherman CD, Berindan-Neagoe I (2012). TNF-alpha gene knockout in triple negative breast cancer cell line induces apoptosis. Int. J. Mol. Sci..

[92] Bilir C, Engin H, Can M, Likhan S, Demirtas D, Kuzu F (2015). Increased serum tumor necrosis factor receptor-associated factor-6 expression in patients with non-metastatic triple-negative breast cancer. Oncol. Lett..

[93] Li HH, Zhu H, Liu LS, Huang Y, Guo J, Li J (2015). Tumour necrosis factor-alpha gene polymorphism is associated with metastasis in patients with triple negative breast cancer. Sci. Rep..

[94] Li CW, Xia W, Huo L, Lim SO, Wu Y, Hsu JL (2012). Epithelial-mesenchymal transition induced by TNF-alpha requires NF-kappaB-mediated transcriptional upregulation of Twist1. Cancer Res..

[95] Habanjar O, Bingula R, Decombat C, Diab-Assaf M, Caldefie-Chezet F, Delort L (2023). Crosstalk of inflammatory cytokines within the breast tumor microenvironment. Int. J. Mol. Sci..

[96] Xu T, Liu J, Xia Y, Wang Z, Li X, Gao Q (2021). Integrated analysis reveals the participation of IL4I1, ITGB7, and FUT7 in reshaping the TNBC immune microenvironment by targeting glycolysis. Ann. Med..

[97] Wu L, Zhang Y, Zheng C, Zhao F, Lin Y (2023). GEMIN4, a potential therapeutic targets for patients with basal-like subtype breast cancer. BMC Womens Health.

[98] Liu Q, Song X, Liu Z, Yu Z (2021). Investigation of candidate genes and pathways in basal/TNBC patients by integrated analysis. Technol. Cancer Res. Treat..

[99] Yang S, Gao W, Wang H, Zhang X, Mi Y, Ding Y (2023). Role of PAX2 in breast cancer verified by bioinformatics analysis and in vitro validation. Ann. Transl. Med..

[100] Kaymak A, Sayols S, Papadopoulou T, Richly H (2018). Role for the transcriptional activator ZRF1 in early metastatic events in breast cancer progression and endocrine resistance. Oncotarget.

[101] Tajadura-Ortega V, Gambardella G, Skinner A, Halim A, Van Coillie J, Schjoldager KTG (2021). O-linked mucin-type glycosylation regulates the transcriptional programme downstream of EGFR. Glycobiology.

[102] Lehmann R, Lee CM, Shugart EC, Benedetti M, Charo RA, Gartner Z (2019). Human organoids: A new dimension in cell biology. Mol. Biol. Cell.

[103] Andrews, S. FastQC: A quality control tool for high throughput sequence data [Online]. Available online at https://www.bioinformatics.babraham.ac.uk/projects/fastqc/ (2010).

[104] Kim D, Paggi JM, Park C, Bennett C, Salzberg SL (2019). Graph-based genome alignment and genotyping with HISAT2 and HISAT-genotype. Nat. Biotechnol..

[105] Trapnell C, Williams BA, Pertea G, Mortazavi A, Kwan G, van Baren MJ (2010). Transcript assembly and quantification by RNA-Seq reveals unannotated transcripts and isoform switching during cell differentiation. Nat. Biotechnol..

[106] Liao Y, Smyth GK, Shi W (2014). featureCounts: An efficient general purpose program for assigning sequence reads to genomic features. Bioinformatics.

[107] Love MI, Huber W, Anders S (2014). Moderated estimation of fold change and dispersion for RNA-seq data with DESeq2. Genome Biol..

[108] Benjamini Y, Hochberg Y (1995). Controlling the false discovery rate: A practical and powerful approach to multiple testing. J. R. Stat. Soc. Ser. B (Methodol.).

[109] Durinck S, Spellman PT, Birney E, Huber W (2009). Mapping identifiers for the integration of genomic datasets with the R/Bioconductor package biomaRt. Nat. Protoc..

[110] Keenan AB, Torre D, Lachmann A, Leong AK, Wojciechowicz ML, Utti V (2019). ChEA3: transcription factor enrichment analysis by orthogonal omics integration. Nucleic Acids Res..

[111] Langfelder P, Horvath S (2008). WGCNA: An R package for weighted correlation network analysis. BMC Bioinform..

[112] Consortium GT (2013). The genotype-tissue expression (GTEx) project. Nat. Genet..

[113] Steentoft C, Vakhrushev SY, Joshi HJ, Kong Y, Vester-Christensen MB, Schjoldager KT (2013). Precision mapping of the human O-GalNAc glycoproteome through SimpleCell technology. EMBO J..

