# Supplementary material for: Truncated O-glycosylation in metastatic triple-negative breast cancer reveals a gene expression signature associated with extracellular matrix and proteolysis
Source: Sci Rep. 2024 Jan 20;14:1809. doi: 10.1038/s41598-024-52204-2 (PMC10799929; doi:10.1038/s41598-024-52204-2)

Supplementary Figures

Truncated O-glycosylation in metastatic triple-negative breast cancer reveals a gene expression signature associated with extracellular matrix and proteolysis

María Florencia Festari, Eugenio Jara, Monique Costa, Andrés Iriarte, Teresa Freire

Supplementary Figure 1

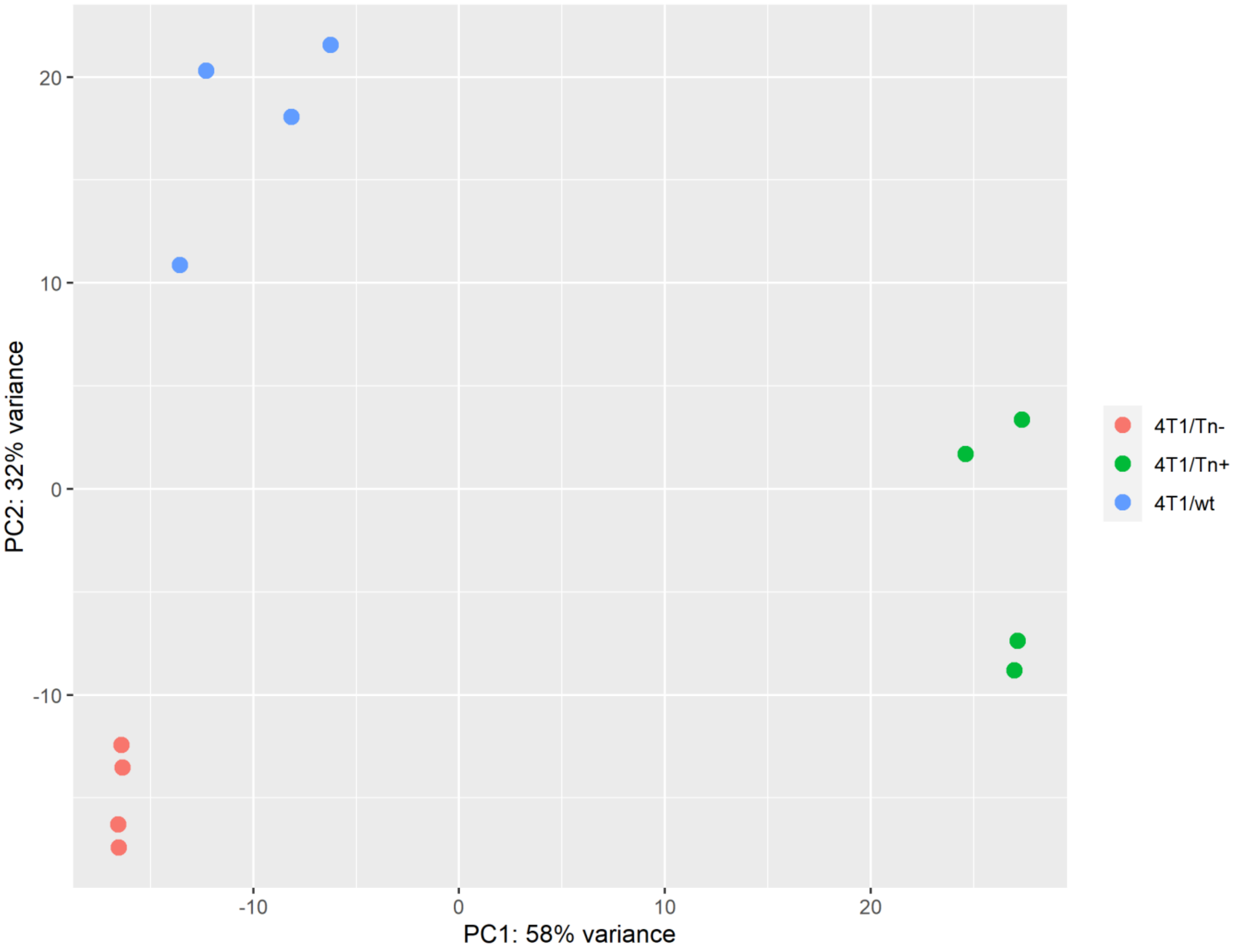

Supplementary Figure 1. Principal component analysis (PCA) plot. The position of samples in the two generated main components is plotted showing the relative similarities in the transcriptomic profile between samples. Variability explained by the first and second components of the PCA is indicated.

Supplementary Figure 2

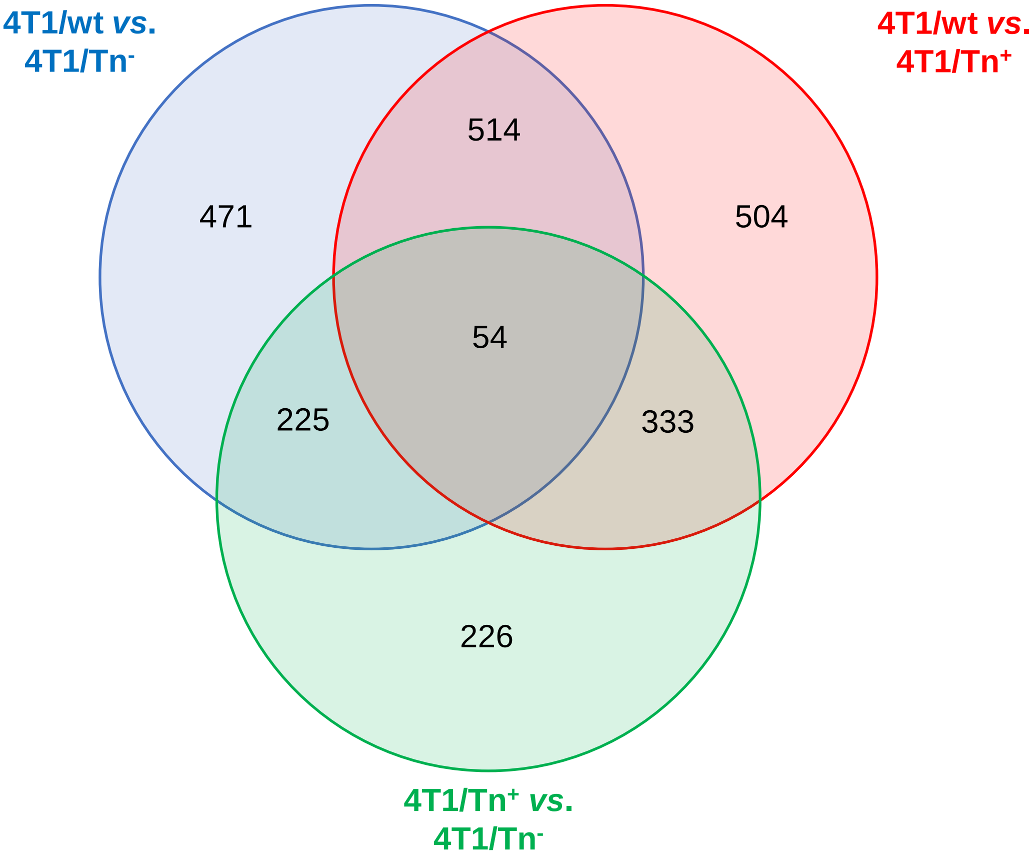

Supplementary Figure 2. Differentially expressed genes (DEGs) of tumor cell lines. Venn diagram indicates shared and unique DEGs identified among pairwise comparisons of analyzed cell lines.

Supplementary Figure 3

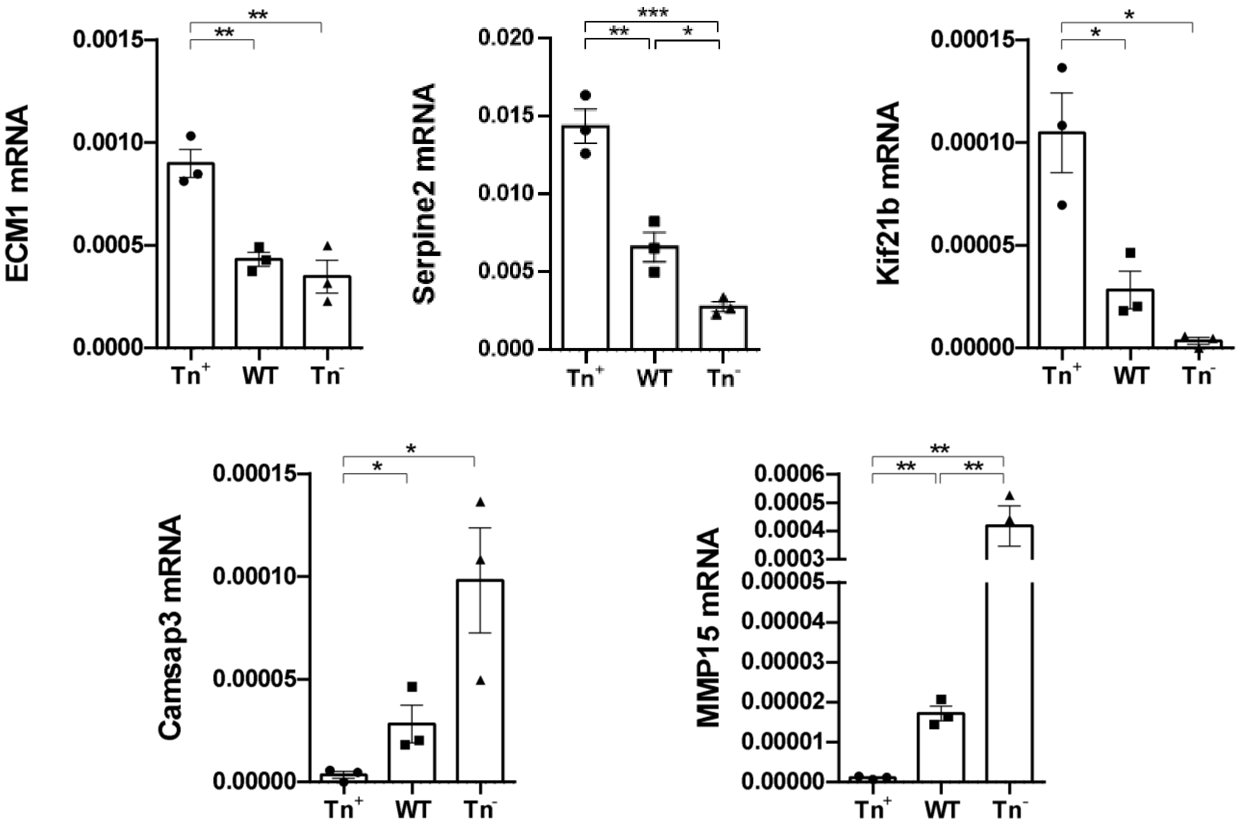

Supplementary Figure 3. Validation of RNAseq analyses by qRT-PCR. *Ecm1, Serpine2, Kif21b, Camsap3* and *Mmp15* gene expression in 4T1/wt, 4T1/Tn^{+} and 4T1/Tn^{-} cells were evaluated by qRT-PCR. mRNA levels were analyzed by qRT-PCR with respect to *Gapdh* expression in 4T1/wt, 4T1/Tn^{+} and 4T1/Tn^{-} cells. Asterisks indicate significant differences (*$p < 0.05$, **$p < 0.01$, ***$p < 0.001$) performed by the Student's unpaired *t*-test.

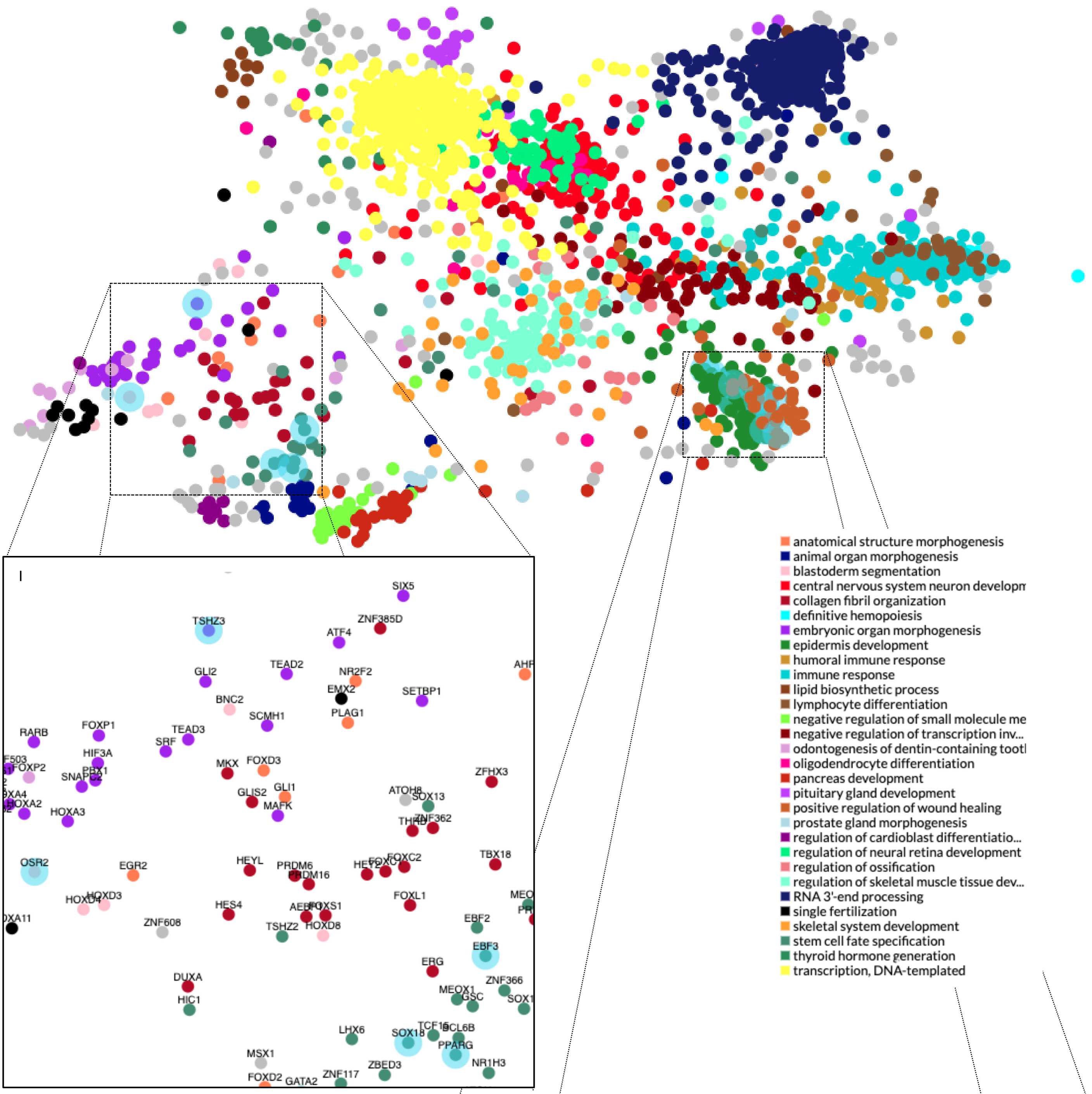

Supplementary Figure 4. Go terms associated to transcription factors (TF). Functional GO terms associated to the TFs projected on the co-expression similarity network of Figure 5 generated in the ChEA3 server. The GTEx modules are colored by the top GO enrichment terms.

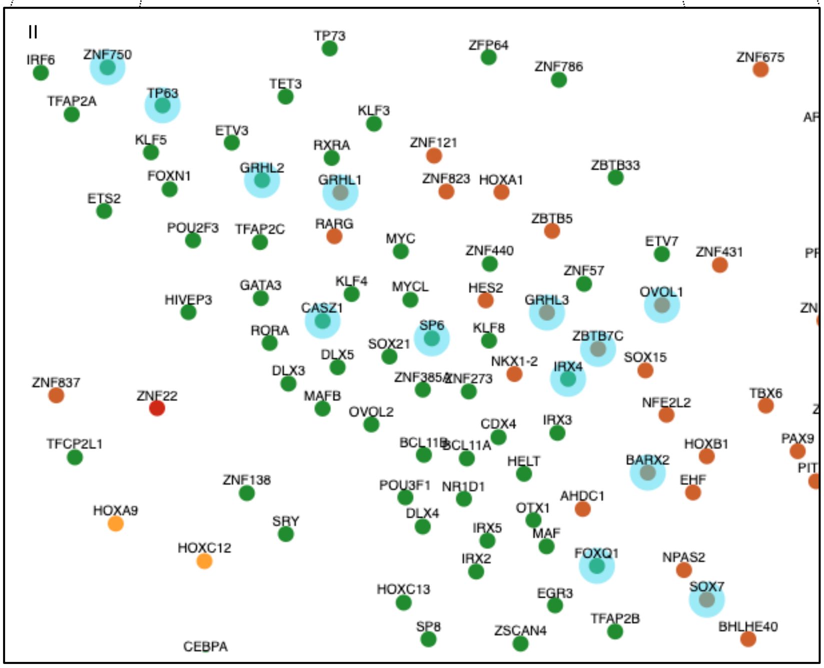

Supplement: Supplementary file 1 — Supplementary Figures. [file 41598_2024_52204_MOESM1_ESM.pdf]
